# Neutrophil extracellular traps in the host defense against sepsis induced by *Burkholderia pseudomallei* (melioidosis)

**DOI:** 10.1186/s40635-014-0021-2

**Published:** 2014-09-03

**Authors:** Hanna K de Jong, Gavin CKW Koh, Ahmed Achouiti, Anne J van der Meer, Ingrid Bulder, Femke Stephan, Joris JTH Roelofs, Nick PJ Day, Sharon J Peacock, Sacha Zeerleder, W Joost Wiersinga

**Affiliations:** Center for Experimental and Molecular Medicine (CEMM), Academic Medical Center, Meibergdreef 9, Room G2-132, Amsterdam, 1105 AZ The Netherlands; Department of Medicine, Division of infectious diseases, Academic Medical Center, Meibergdreef 9, Room G2-132, Amsterdam, 1105 AZ The Netherlands; Department of Medicine, Addenbrooke’s Hospital, University of Cambridge, Cambridge, CB2 0QQ UK; Mahidol-Oxford Tropical Medicine Research Unit, Faculty of Tropical Medicine, Mahidol University, Bangkok, 10400 Thailand; Department of Infection and Tropical Medicine, Heartlands Hospital, Birmingham, B9 5SS UK; Department of Immunopathology, Sanquin Research, Amsterdam, 1066 CX The Netherlands; Department of Pathology, Academic Medical Centre, Amsterdam, 1105 AZ The Netherlands; Nuffield Department of Clinical Medicine, Churchill Hospital, University of Oxford, Oxford, OX3 7LJ UK; Department of Haematology, Academic Medical Centre, Amsterdam, 1105 AZ The Netherlands

**Keywords:** Sepsis, *Burkholderia pseudomallei*, Melioidosis, Neutrophil extracellular traps (NETs), Neutrophils, Diabetes, Survival, DNase, Innate immunity

## Abstract

**Background:**

Neutrophil extracellular traps (NETs) are a central player in the host response to bacteria: neutrophils release extracellular DNA (nucleosomes) and neutrophil elastase to entrap and kill bacteria. We studied the role of NETs in *Burkholderia pseudomallei* infection (melioidosis), an important cause of Gram-negative sepsis in Southeast Asia.

**Methods:**

In a prospective observational study, circulating nucleosomes and neutrophil elastase were assayed in 44 patients with Gram-negative sepsis caused by *B. pseudomallei* (melioidosis) and 82 controls. Functional assays included human neutrophil stimulation and killing assays and a murine model of *B. pseudomallei* infection in which NET function was compromised using DNase. Specified pathogen-free 8- to 12-week-old C57BL/6 mice were sacrificed post-infection to assess bacterial loads, inflammation, and pathology.

**Results:**

Nucleosome and neutrophil elastase levels were markedly elevated in patients compared to controls. NETs killed *B. pseudomallei* effectively, and neutrophils stimulated with *B. pseudomallei* showed increased elastase and DNA release in a time- and dose-dependent matter. In mice, NET disruption with intravenous DNase administration resulted in decreased nucleosome levels. Although DNase treatment of mice resulted in diminished liver inflammation, no differences were observed in bacterial dissemination or systemic inflammation.

**Conclusion:**

*B. pseudomallei* is a potent inducer of NETosis which was reflected by greatly increased levels of NET-related components in melioidosis patients. Although NETs exhibited antibacterial activity against *B. pseudomallei*, NET formation did not protect against bacterial dissemination and inflammation during *B. pseudomallei*-induced sepsis.

**Electronic supplementary material:**

The online version of this article (doi:10.1186/s40635-014-0021-2) contains supplementary material, which is available to authorized users.

## Background

Melioidosis (Gram-negative infection caused by *Burkholderia pseudomallei*) is a major cause of severe community-acquired sepsis in Southeast Asia and northern Australia [1,2]. The clinical manifestations of *B. pseudomallei* infection range from chronic skin abscess to acute fulminant sepsis. Despite antibiotic treatment, melioidosis patients with bacteremia or pneumonia have a mortality rate of approximately 40% [3,1,2]. Melioidosis is therefore a good clinical model in which to study Gram-negative sepsis [4-6].

Neutrophils constitute the central line of the innate immune defense against many bacteria [7,8]. Their arsenal is impressive and ranges from killing and phagocytosis to the production and release of antimicrobials and immunoregulatory cytokines [9,2]. Neutrophils play a critical role in the host defense against *B. pseudomallei*. Although excessive neutrophil recruitment may be detrimental to the host [10], neutrophils are essential for early bacterial containment. Activated neutrophils are rapidly recruited to the lungs upon infection, and neutrophil depletion leads to accelerated mortality in mice [11,12]. Not surprisingly, conditions that predispose individuals to melioidosis, most notably diabetes mellitus, are associated with impaired neutrophil function [3,13-15].

Neutrophil extracellular traps (NETs; another effector mechanism of neutrophils) consist of extracellular DNA and histone-based structures decorated by antimicrobials such as neutrophil elastase and myeloperoxidase. They ensnare bacteria, degrade virulence factors, and ultimately kill their target [16]. Besides their antimicrobial function, NETs also induce a strong procoagulant response via extracellular nucleosomes (extracellular frames consisting of DNA and histones) within the NETs, which stimulates the proteolytic activity of neutrophil elastase and in turn promotes coagulation [17-20]. Virtually, all microbes that cause sepsis (including *B. pseudomallei*) have been shown to induce NET formation *in vitro* [7,21,16]. In a mouse model of intraperitoneal *Escherichia coli* infection, it was recently shown that NETs released into the vasculature were able to trap bacteria from the bloodstream and prevent bacterial dissemination [22]. Intriguingly, some bacteria (such as certain strains of *Streptococcus pneumoniae* and *Pseudomonas aeruginosa*) have developed mechanisms to circumvent NET-mediated killing, for example, via DNase [21,23,8,19]. In theory, overwhelming NETosis or a reduced clearance capacity of NETs could be detrimental to the host during sepsis and contribute to ongoing inflammation, organ damage, and/or exhaustion of the immune system [21]. In the context of sepsis, free circulating DNA is regarded as an endogenous danger signal or ‘danger-associated molecular pattern’ (DAMP). DAMPs are released during inflammatory stress and trigger the host immune response [24-26]. The role and consequences of NET formation during human sepsis, however, remain ill-defined.

In this study, we examined the expression of NET-related markers in patients with melioidosis. We examined next the functional role of NETs in an *in vitro* model of *B. pseudomallei* infection, then examined the role of NETs *in vivo* by treating mice infected with *B. pseudomallei* with DNase to disrupt the DNA backbone of NETs.

## Methods

### Studies of clinical melioidosis

The clinical study was approved by the Oxford Tropical Research Ethics Committee (OXTREC 018-07) and the Ethics Committee of Mahidol University (MUTM 2008-001-01). This co-host has been described previously [27]: 44 patients with culture-proven melioidosis and sepsis, from whom ethylenediaminetetraacetic acid (EDTA) plasma was obtained on day of recruitment, 7 days after, and at a follow-up clinic ≥28 days after discharge. Patients were recruited within 48 h of admission. All patients had culture-proven melioidosis and had two out of four criteria for systemic inflammatory response syndrome (SIRS) [28]. Eligible patients had received active antimicrobial chemotherapy for less than 48 h (ceftazidime, amoxicillin-clavulanate, meropenem, or imipenem). Thirty-four patients were classed as diabetic if they had an HbA1c ≥7.8% at enrollment or a diagnosis of diabetes made prior to admission. Eighty-two healthy subjects served as controls (52 with diabetes and 30 without; Additional file 1: Table S1).

Peripheral leukocyte gene expression was determined via microarray analysis, as described previously [29]. RNA was assayed using HumanWG-6 v3.0 Expression BeadChips (Illumina®, Illumina Inc., San Diego, CA, USA), and data have been deposited at ArrayExpress, EMBL-EBI (accession-number E-TABM-852-n). We interrogated this database for proteins co-localizing to NETs [17]. To assess human neutrophil elastase, complexes together with α1-antitrypsin were assayed in plasma by ELISA, as elastase in plasma is immediately inactivated by the formation of these covalent complexes and is hardly detectable in its active form [30,31]. Nucleosome levels were determined in EDTA plasma by enzyme-linked immunosorbent assay (ELISA) [32,33]. In brief, the capture antibody was CLB-ANA/60 (which recognizes histone-3). The detector antibody was biotinylated CLB-ANA/58 (which recognizes an epitope exposed on complexes of histone-2A, histone-2B, and dsDNA). The reaction was read using poly-HRP and 3,3′,5,5′-tetramethylbenzidine as the chromogenic substrate.

### Human neutrophil stimulation

*B. pseudomallei* strain 1026b [34] was cultured overnight and transferred to fresh Luria broth for 3 h in a 37°C shaker to yield bacteria at mid log-phase*. Staphylococcus aureus* strain LAC (USA 300; a known NET inducer [35]) was grown to stationary phase for 20 h in tryptic soy and brain-heart infusion broth at 37°C. Bacteria were centrifuged for 2 min at 300×*g* and the resulting pellet resuspended in Hanks' balanced salt solution (HBSS) with Ca^2+^ and Mg^2+^ (HBSS+/+; Gibco, Invitrogen, Carlsbad, CA, USA) before use.

Human neutrophils were isolated from peripheral blood of healthy donors using Polymorphprep™ (Axis-Shield, Oslo, Norway). Following erythrocyte lysis, neutrophils were washed and resuspended with HBSS without Ca^2+^ and Mg^2+^ (HBSS−/−) [7]; ≥99% of neutrophils were viable as determined by trypan blue exclusion [36]. Human NETs were induced as described previously [7,17]. In brief, neutrophils were resuspended in HBSS−/− (final concentration 2 × 10^5^ cells/100 μL) and infected with 100 μL HBSS+/+ containing live *B. pseudomallei* (final concentration 2 × 10^5^ to 2 × 10^7^ cfu) at 37°C and 5% CO_2_ to induce NET formation. HBSS+/+ medium alone served as the negative control; 20 nM phorbol 12-myristate 13-acetate (PMA; Sigma-Aldrich Corporation, St. Louis, MO, USA) and *S. aureus* (2 × 10^5^ cfu) served as positive controls. Supernatant was harvested at 0, 1, and 4 h and passed through a 0.2-μm filter (Millipore, Billerica, MA, USA) to remove live bacteria prior to storage at −20°C. For DNA quantification, cells were washed once with medium before adding HBSS−/−, Ca^2+^ and Mg^2+^ (5 mM), and 0.1 U/mL DNase (Roche Diagnostics, Indianapolis, IN, USA) and incubated for a further 2 h at 37°C. Sterile 20 mM EDTA (Thermo Scientific, Waltham, MA, USA) was added to stop DNase activity. In control wells, EDTA was added prior to incubation with DNase.

To examine the growth-inhibiting properties of NETs on *B. pseudomallei*, 3 × 10^5^ human neutrophils per well were resuspended in 50 μL Iscove's modified Dulbecco's medium (IMDM; Gibco) in a 96-well plate, then stimulated with 100 nM PMA for 5 h at 37°C (5% CO_2_) to induce NET formation [7]. Following NET formation, the cells were preincubated for 30 min at 37°C with 50 U DNase, heat-inactivated DNase (80°C for 10 min), or phosphate-buffered saline (PBS). NETs were stained with Sytox Green 5 mM (Molecular Probes, Eugene, OR, USA) and then visualized using a confocal microscope (Olympus IX81, Olympus Corporation, Tokyo, Japan). The supernatant was discarded, and approximately 7 × 10^2^ cfu/100 μL IMDM of log-phase *B. pseudomallei* were added to the wells and spun down for 5 min at 300×*g*. After 10 h, bacterial loads were quantified by culture on horse blood agar.

Free elastase in the supernatant was assayed by ELISA as in the absence of plasma no complexes are formed with α1-antitrypsin, as described previously [37,30]. Since NET formation is quantified by the measurement of DNA release after DNase treatment, and DNA will degrade the epitope of the anti-nucleosome antibody, measurement of nucleosomes by this ELISA is not suitable [38]. Therefore extracellular DNA was quantified in the supernatant using a Quant-iT™ Picogreen® dsDNA Assay Kit (Invitrogen) according to the manufacturer's instructions and was analyzed with a SpectraFluor Plus absorbance reader (Tecan Group, Männedorf, Switzerland).

### Mouse experiments

Mouse experiments were approved by the Academic Medical Center Animal Use and Welfare Committee (DIX16AB). Pathogen-free 8- to 12-week-old C57BL/6 mice (Charles River, Wilmington, MA, USA) were inoculated intranasally with 1,000 (LD_100_) or 125 (LD_50_) cfu *B. pseudomallei* 1026b in a 50-μL saline solution (eight per group) as described previously [39,40]. Mice were treated intravenously with 2,000 U DNase I (Roche) in 200 μL PBS at 0, 4, 20, 26, 42, and 50 h post-inoculation to disrupt the NET backbone; control mice received 200 μL PBS intravenously as described [22]. Lung, broncheoalveolar lavage fluid (BALF), liver, and EDTA blood were collected for bacterial culture and cytokine assays as described previously [41]. Nucleosomes were assayed in plasma and BALF to quantify NET formation.

Neutrophil counts in BALF were determined by FACS (FACSCalibur; Becton Dickson, San Jose, CA, USA) using directly labeled antibodies against Gr-1 (Ly-6G; Gr-1 FITC; BD Pharmingen, BD Biosciences, San Jose, USA). Nucleosome levels were measured by ELISA [32,33]. Tumor necrosis factor (TNF)-α, interleukin (IL)-6, IL-10, and chemokine (C-X-C motif) ligand 1 (CXCL1 or KC) in lung homogenates were measured by sandwich ELISA (R&D Systems, Minneapolis, MN, USA) [41]. Plasma cytokine concentrations (TNF-α, IL-6, interferon (IFN)-γ, IL-10, monocyte chemotactic protein-1 [MCP-1 or CCL2], and IL-12p70) were measured by cytometric bead array (BD Biosciences). Lactate dehydrogenase (LDH), aspartate aminotransferase (AST), alanine transaminase (ALT), urea, and creatinine were measured in plasma with spectrophotometry (Roche Diagnostics). Lung and liver inflammation were scored histologically as described previously [42,41,40]. In brief, lung inflammation and damage of tissue were analyzed with respect to the following parameters: surface with pneumonia, necrosis and/or formation of abscess, interstitial inflammation, endothelialitis, bronchitis, edema, thrombus formation, and pleuritis. Liver pathology was analyzed with respect to the following parameters: area of liver with parenchymal inflammation, necrosis and/or abscess formation, portal inflammation, and thrombus formation.

### Statistical analysis

Gene expression data were analyzed using R-Bioconductor, as described previously [29]. We log-transformed neutrophil elastase and nucleosome concentrations to correct for heteroscedasticity. For correlations, Pearson *r* is reported. In the kinetic assay, *P* values reported are for the slope of the linear regression model with elastase or DNA as the dependent variable and time point or stimulus as independent variables. Differences between murine groups were analyzed by the Mann-Whitney *U* or unpaired *t* test where appropriate. Analyses were done in GraphPad Prism version 6.0 for Mac OS X (GraphPad Software), and data are expressed as mean and SD unless stated otherwise.

## Results

### Nucleosome and neutrophil elastase levels are markedly elevated in patients with melioidosis and correlate with disease severity

**Figure 1:**
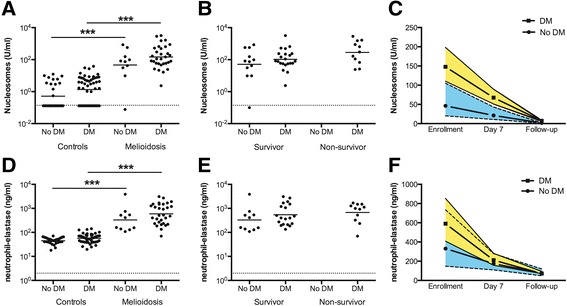
**NET-related markers are abundantly present in plasma from patients with melioidosis with or without diabetes.** Increased levels of NET-related markers such as nucleosomes and neutrophil elastase are measured in plasma of patients on day of initial presentation with sepsis caused by *B. pseudomallei* (melioidosis) compared to healthy control subjects. No difference was found between melioidosis patients with or without diabetes **(A,** **D)**. No difference was seen between melioidosis survivors versus non-survivors **(B,** **E)**. Straight lines represent the mean, and dashed lines mark the lower limit of detection. Nucleosome levels and neutrophil elastase were measured in all surviving patients on day of enrollment, day 7, and day 28 (follow-up). There is no difference when looking at the normalization of the NET-related makers over time between melioidosis patients with or without diabetes **(C,** **F)**. Mean and SEM are shown. ***P < 0.001 (unpaired *t* test).

To establish the presence of NETs during clinical melioidosis, we measured nucleosomes (complexes of extracellular DNA and histones and a useful measure of cell-free DNA in plasma) and neutrophil elastase (a measure of neutrophil degranulation) in the plasma of 44 patients with septic melioidosis and 82 healthy controls. Both are key NET-related markers [17,18,43], and both nucleosome (*P* < 0.0001) and elastase levels (*P* < 0.0001) were abundant in the plasma of patients with melioidosis on day of recruitment compared to healthy controls (Figure 1A,D).

**Figure 2:**
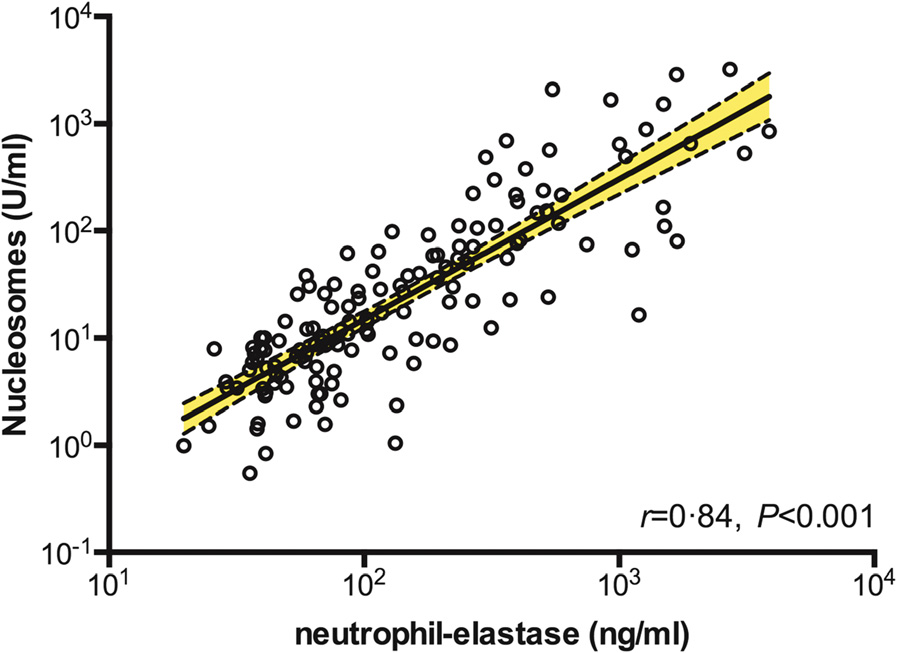
**Neutrophil elastase and nucleosome release are highly correlated in patients with melioidosis.** Linear regression of neutrophil elastase versus nucleosomes. Correlation coefficient reported is for Pearson's *r*.

The mortality rate of 27% (12 of 44) in this cohort enabled us to correlate the presence of these NET-related markers with disease severity. No differences were seen in the admission nucleosome and neutrophil elastase levels when comparing survivors and non-survivors (Figure 1B,E). No differences were seen in concentration levels of both NET markers in non-survivors on day of enrollment and day 7. All non-survivors died before the 28 day time point. However, a marked difference in nucleosome concentrations between survivors and non-survivors at day 7 was seen: at this time point, patients that went on to die had significantly higher nucleosome levels (192.3 ± 5) compared to survivors (33.6 ± 4; *P =* 0.001). Evidence for an association between nucleosome and neutrophil elastase plasma levels and disease severity was obtained in patients who survived, a second blood sample was drawn during treatment (at day 7) and after successful completion of therapy (≥28 days from discharge). In all surviving patients, plasma nucleosome and elastase concentrations moved toward normal (Figure 1C,F). Moreover, neutrophil activation (evidenced by elastase-α_1_-antitrypsin complexes) was strongly correlated with nucleosome levels at admission (*P* < 0.001, Figure 2), supporting the hypothesis that neutrophils are the source of nucleosomes. Of note, gene expression of proteins known to be associated with NET formation [17] was determined in total peripheral leukocytes derived from patients with culture-confirmed septic melioidosis and healthy controls. In line with the protein measurements, a marked up-regulation of genes encoding the central NET-associated proteins nucleosome histone-2B and histone-4 was found (Additional file 2: Table S2).

### No influence of diabetes on the release of the NET-related markers

Since diabetes is the main risk factor for melioidosis and diabetes is known to negatively influence neutrophil function [3,15,14], we next sought to determine whether the presence of diabetes influenced the release of NET-related markers in our cohort of patients of whom 72% were diabetic. We found no difference in the release of the NET-related markers in melioidosis when comparing patients with or without diabetes on day of recruitment. In addition, NET-related markers were not different in healthy controls with or without diabetes (Figure 1A,D).

### Elastase and extracellular DNA are released by neutrophils in a dose- and time-dependent fashion upon stimulation with *B. pseudomallei*

**Figure 3:**
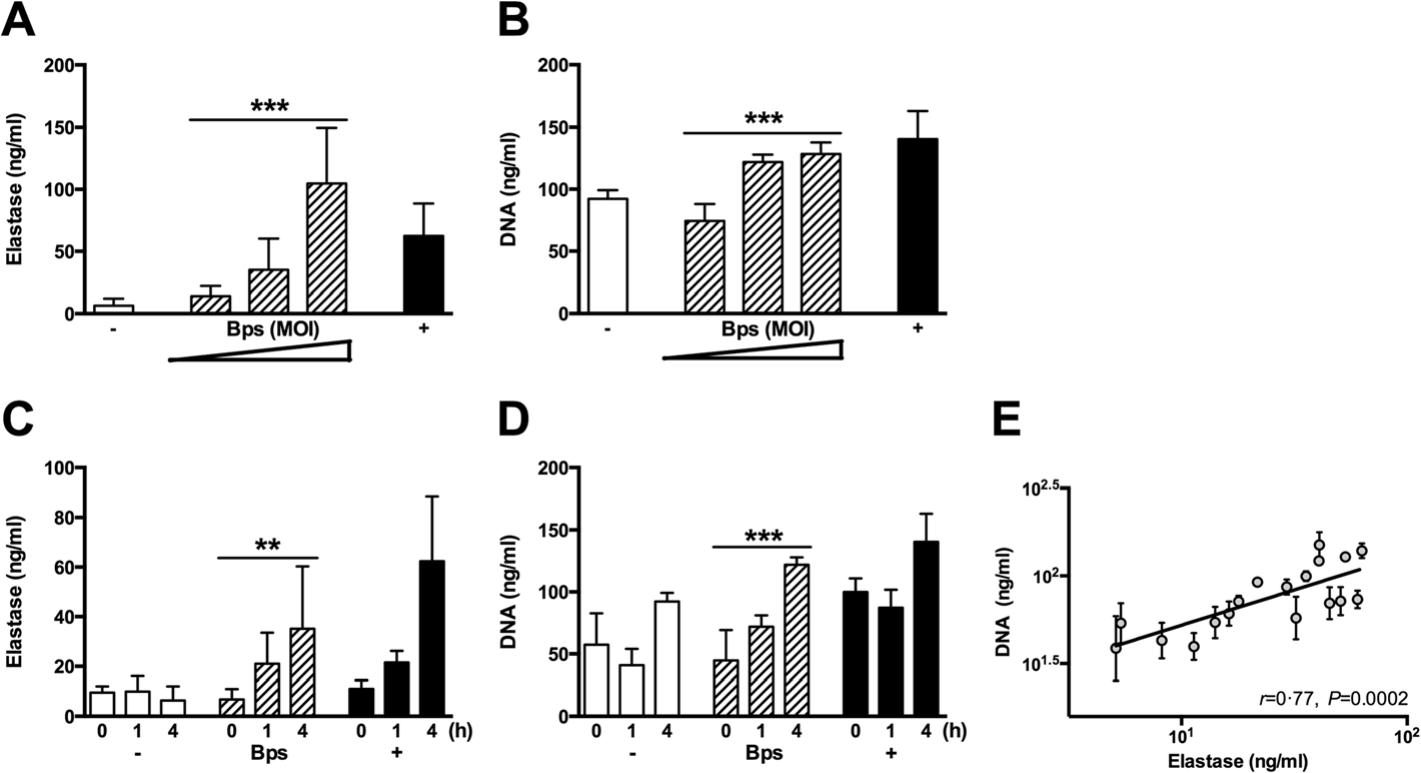
**NET-related markers are released by neutrophils in a dose- and time-dependent fashion.** The kinetics of *B. pseudomallei*-induced NET formation at various multiplicities of infections (MOI; 10°, 10^1^, 10^2^) and time points (0, 1, and 4 h) were assessed by quantifying elastase **(A,** **C)** and extracellular DNA using a Picogreen dsDNA kit **(B,** **D)**, in the supernatant of stimulated isolated human neutrophils. The negative control (−) was medium (HBSS−/−), and the positive control (+) was PMA, a known inducer of NETs. Both elastase and DNA increase in a time- and dose-dependent manner following *B. pseudomallei* (Bps) stimulation*.* The mean and SD are expressed. **(E)** The two NET markers, elastase and DNA, were highly correlated, suggesting that neutrophils are the source of this extracellular DNA. All experiments used whole blood from three to six different healthy human volunteers. Elastase results were log-transformed to correct for heteroscedasticity. *P* values reported are for the slope of linear regression models with elastase or DNA as the dependent variables and time point or MOI as independent variables. **P < 0.01; ***P < 0.001.

To examine whether neutrophils were the common source of NET-related marker release in patients with melioidosis, we isolated human neutrophils from healthy individuals and stimulated them with various known NET inducers as well as *B. pseudomallei* upon which the kinetics of elastase and extracellular DNA in culture supernatants was determined. Consistent with the patient data, elastase levels increased following stimulation with *B. pseudomallei*, and this occurred in a dose-dependent (*P* < 0.001, Figure 3A) and time-dependent manner (*P =* 0.005, Figure 3C). Extracellular DNA release also increased in a time- and dose-dependent manner following *B. pseudomallei* infection: extracellular DNA concentrations increased as the multiplicity of infection (MOI) increased (*P* < 0.001, Figure 3C) and over time (*P* < 0.001, Figure 3D). Of note, *B. pseudomallei*-infected cells released similar amounts of elastase and DNA as *S. aureus* (Additional file 3: Figure S1), itself a potent NET inducer [44]. Elastase levels and DNA release were also strongly correlated, reflecting the clinical data (Figure 3E).

### NETs exhibit antibacterial activity against *B. pseudomallei*

**Figure 4:**
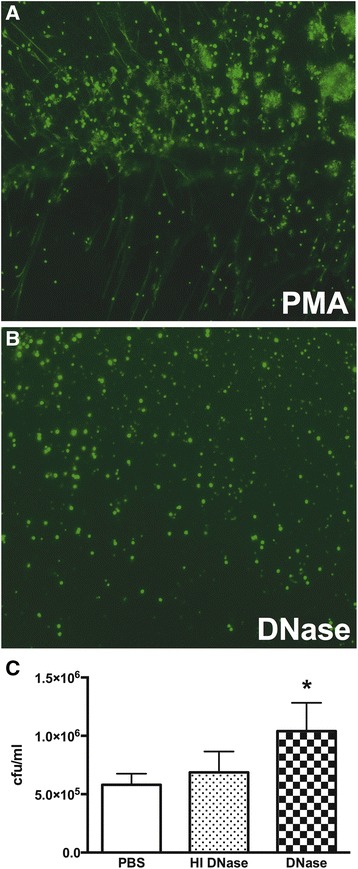
**NETs exhibit antibacterial activity against** ***B. pseudomallei.*** Representative confocal micrographs of human neutrophils stimulated with PMA **(A)** to produce NETs and post-stimulation treated with PBS, heat-inactivated (HI) DNase, or active DNase **(B)**, before infection with 7 × 10^2^ cfu *B. pseudomallei* **(C)**. Data are expressed as mean and SD. *P* value was determined via one-way ANOVA. Blood from three different healthy human volunteers was used.

We next sought to examine whether activated NETs were able to kill *B. pseudomallei*. We pre-stimulated human neutrophils with PMA for 5 h to induce NET formation [7,17], then preincubated the cells with PBS or DNase prior to infection with *B. pseudomallei.* Extracellular degradation of DNA by DNase treatment was confirmed by confocal microscopy (Figure 4A,B). Disrupting the backbone of the NETs with active DNase impaired the killing of *B. pseudomallei* by NETs from human neutrophils (Figure 4C). Taken together, these data confirm the involvement of NETs in the killing of *B. pseudomallei* by neutrophils.

### NETs do not protect against bacterial dissemination in *B. pseudomallei*-induced sepsis

**Figure 5:**
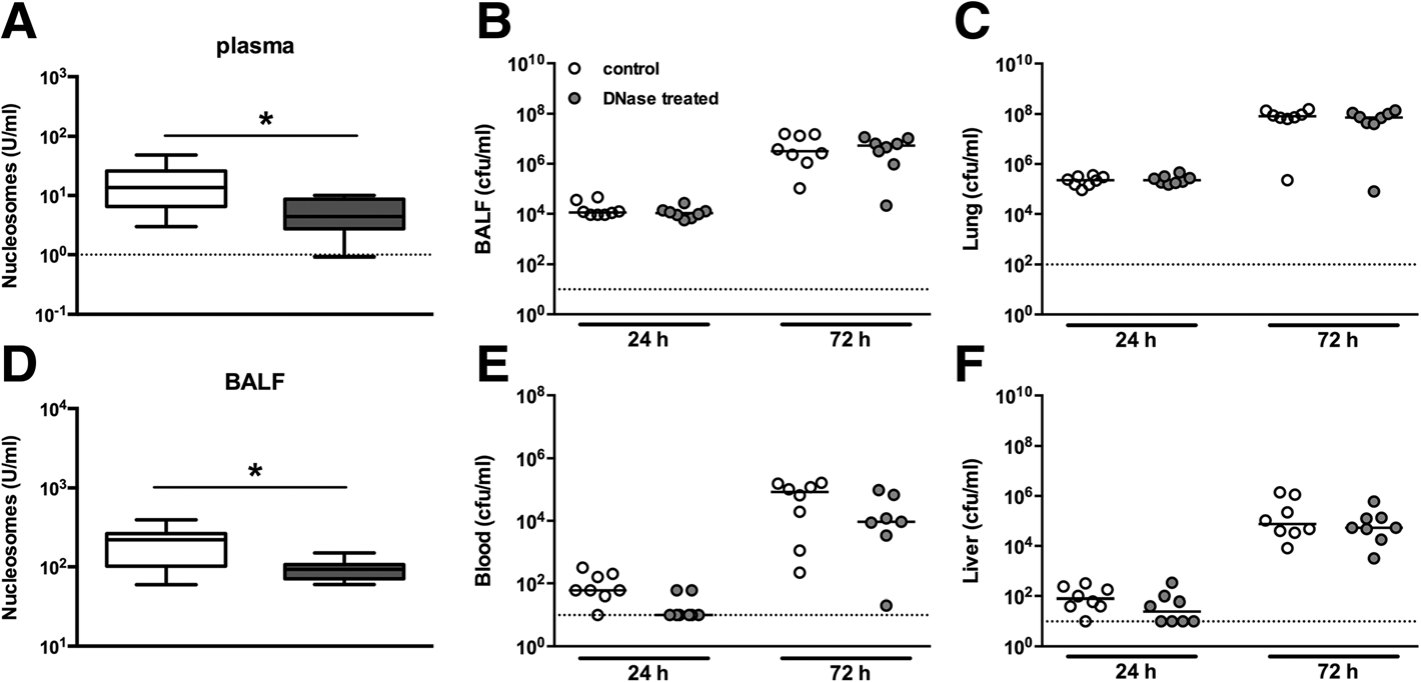
**Disruption of NETs does not influence bacterial dissemination during experimental melioidosis.** C57/Bl6 wild-type (WT) mice were intranasal infected with *B. pseudomallei* (10^3^ cfu) and either treated with intravenous PBS (control group) or 2,000 U DNase I recombinant (DNase-treated group) at various time points [22]. Compared to control mice, DNase-treated mice show reduced nucleosome levels in plasma **(A)** and bronchoalveolar lavage fluid (BALF) **(D)** after 24 h of infection. Data are expressed as box-and-whisker diagrams depicting the smallest observation, lower quartile, median, upper quartile, and largest observation. Equal bacterial titers were seen in BALF **(B)**, lung homogenates **(C)**, blood **(E)**, and liver homogenates **(F)** during all time points after infection in the DNase I-treated mice compared to control mice. Median values (straight lines) and the lower limit of detection (dashed lines) are shown. Graphs depict eight mice per group and time point. Statistical significance was determined using the unpaired Mann-Whitney *U* test. *P < 0.05.

Having confirmed that NETs are capable of killing of *B. pseudomallei in vitro*, we sought to replicate this finding in a murine model of melioidosis. We infected mice with a lethal challenge of *B. pseudomallei* (10^3^ cfu) and treated half of them with intravenous DNase twice daily in order to disrupt NET formation, as described previously [22]. Animals were sacrificed after 24 or 72 h to determine bacterial loads in BALF and lung (primary sites of the infection), and the liver and blood (to evaluate the extent of bacterial dissemination). In this murine model of Gram-negative sepsis, untreated mice showed an increase of nucleosome levels in plasma (17.6 ± 15) and BALF (202.7 ± 110) 24 h post-infection (Figure 5A,D). In comparison, uninfected untreated mice (*t* = 0) had low to undetected plasma and BALF nucleosome levels (5.6 ± 3 and 12.9 ± 5, respectively). DNase treatment resulted in successful disruption of intravascular NET formation, as illustrated by significantly decreased nucleosome levels in both plasma (5.3 ± 3, *P =* 0.03) and BALF (93.9 ± 29, *P =* 0.02) compared to untreated mice 24 h post-infection (Figure 5A,D). Disruption of intravascular NETs did not, however, result in changes in bacterial loads in BALF, lung, blood, or liver (Figure 5). In line with the bacterial loads, no differences were seen in total cell count or neutrophil influx in BALF between DNase-treated and untreated mice after infection (data not shown). Because cytokines are important regulators of the inflammatory response to bacterial pneumonia [45], we measured pulmonary (TNF-α, IL-6, IL-10, CXCL1) and systemic (TNF-α, MCP-1, IL-6, IFN-γ, IL-10, IL12p70) cytokine and chemokine levels (Table 1). Levels of all cytokines were elevated in both pulmonary and systemic compartments (with the exception of IL-10) upon *B. pseudomallei* infection. However, in complete correspondence with bacterial loads and cellular influx, no differences were seen between the DNase-treated animals and controls.

**Table 1:** **Cytokines and markers for systemic organ injury do not differ between mice treated with intravenous DNase or PBS post-infection with** ***B. pseudomallei***

	**24 h**			**72 h**		
	**Control**	**DNase**	***P*** **value**	**Control**	**DNase**	***P*** **value**
Plasma						
TNF-α (pg/mL)	3.3 ± 2	4.1 ± 2	0.41	407 ± 319	328.3 ± 300	0.62
MCP-1 (pg/mL)	11.7 ± 13	21.5 ± 8	0.09	3,976 ± 2,183	2,879 ± 1,937	0.31
IL-12p70 (pg/mL)	ND	ND		8.6 ± 10	21.6 ± 20	0.13
IL-6 (pg/mL)	115.3 ± 73	104 ± 62	0.74	8,022 ± 3,814	6,990 ± 4,252	0.62
IFN-γ (pg/mL)	4.0 ± 6	4.8 ± 4	0.76	1,914 ± 1,152	1,791 ± 1,490	0.86
IL-10 (pg/mL)	ND	ND		ND	ND	
AST (U/L)	63.6 ± 6	74.4 ± 37	0.83	356.6 ± 191	247 ± 143	0.50
ALT (U/L)	21.7 ± 9	27 ± 24	0.92	155.4 ± 59	138 ± 98	0.60
LDH (U/L)	210 ± 74	312.9 ± 157	0.21	1,115 ± 758	873.2 ± 375	0.71
Urea (mmol/L)	9.8 ± 2	7.7 ± 2	0.04	15.9 ± 10	14.4 ± 5	0.91
Creatinine (μmol/L)	11.3 ± 2	9.6 ± 4	0.43	15.7 ± 17	8.4 ± 7	0.53
Lung						
TNF-α (pg/mL)	239.1 ± 110	427.8 ± 100	0.003	3,182 ± 1,440	3,261 ± 1,382	0.91
IL-6 (pg/mL)	945.9 ± 348	1,103 ± 201	0.29	8,601 ± 3,329	7,521 ± 3,482	0.54
IL-10 (pg/mL)	205.8 ± 147	266.4 ± 57	0.29	559.4 ± 219	498.3 ± 107	0.49
CXCL1 (pg/mL)	6,771 ± 4,247	6,147 ± 2,284	0.72	26,421 ± 10,123	26,305 ± 10,450	>0.99

Cytokines and markers for systemic organ injury were measured in plasma and/or lung homogenates 24 and 72 h post-infection. Data are means ± SD (cytokines) or median (range) of five to eight mice per group. Actual *P* values are shown, determined via *t* test or Mann-Whitney *U* where appropriate; ND, not detectable or below detection limit; TNF-α, tumor necrosis factor-alpha; MCP-1, monocyte chemo-attractive protein-1; IL, interleukin; IFN-γ, interferon-gamma; AST, aspartate transaminase; ALT, alanine transaminase; LDH, lactate dehydrogenase; CXCL1, chemokine (C-X-C motif) ligand 1.

We speculated that the infectious challenge might have been too high to reveal an effect of NET blockade in our model and therefore repeated the experiments with a lower inoculum (1.25 × 10^2^ cfu which is the LD_50_). Again, although significant reductions of nucleosomes of DNase-treated mice were seen in BALF (*P =* 0.002) compared to untreated mice, no differences were seen in bacterial burdens in BALF, lung, blood, or liver nor local or systemic inflammatory cytokine levels 72 h post-infection (data not shown). These results indicate that disrupting NET release by degradation of the DNA backbone does not impair the host defense against *B. pseudomallei* infection.

### Intravascular NETs cause collateral liver damage during experimental melioidosis

**Figure 6:**
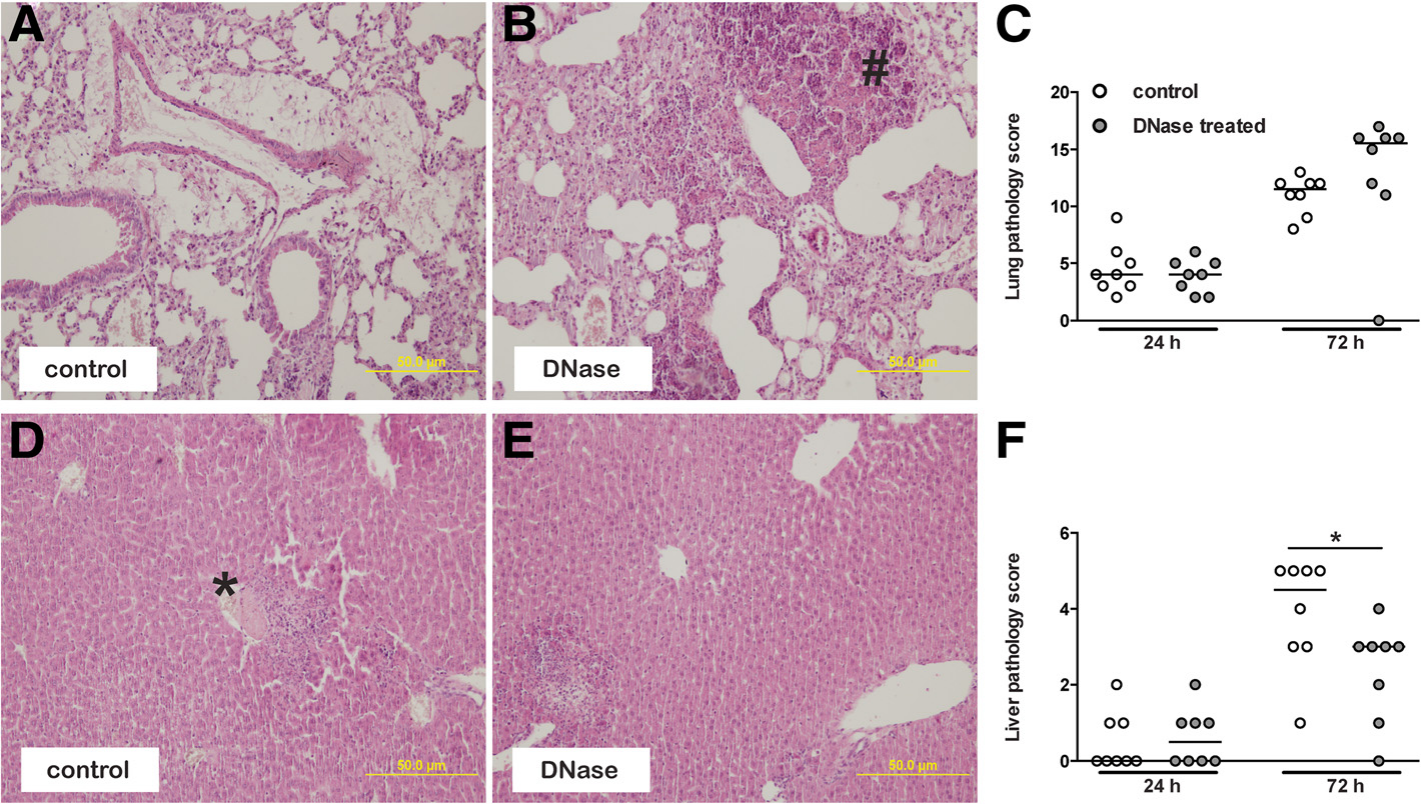
**Effect of DNase treatment on pulmonary and hepatic inflammation after infection with** ***B. pseudomallei.*** Representative hematoxylin and eosin stained slides of lung **(A,** **B)** and liver **(D,** **E)** of control and DNase-treated mice 72 h post-infection with *B. pseudomallei* (10^3^ cfu). Marked inflammation was seen post-infection, and areas of necrosis (marked with a number sign) and thrombosis formation (marked with an asterisk) were seen in both groups. Significantly less liver inflammation was observed in the DNase-treated group when compared to controls 72 h after infection. Total organ pathology score (lung and liver **(C,** **F)**) was determined 24 and 72 h post-infection according to the scoring system as described [41,39,40]. Graphs depict eight mice per group and time point. Median values are shown, and statistical significance was determined using the unpaired Mann-Whitney *U* test. *P < 0.05.

It has been reported previously that NETs are implicated in tissue damage during *E. coli* sepsis [22]. We therefore examined lung and liver tissue histologically for inflammation. No differences in lung pathology scores were observed between groups (Figure 6A,B,C). In accordance with a previous report, however, we found that significantly less liver inflammation was seen in the DNase-treated group when compared to controls at 72 h post-infection (*P =* 0.04, Figure 6D,E,F). It should be noted, however, that serum levels of AST and ALT, both markers of hepatocellular injury, as well as urea, creatinine, and LDH, markers of kidney and systemic organ injury, showed a trend toward lower values in the DNase-treated mice, although this did not reach statistical significance (Table 1). We conclude that there is only a limited role for NETs during *B. pseudomallei* infection *in vivo*.

## Discussion

NETs have emerged as key players in the innate host response to many bacteria. Neutrophils release extracellular DNA (including nucleosomes) and neutrophil elastase to entrap and capture bacteria [16], and animals depleted of neutrophils are markedly more susceptible to *B. pseudomallei* infection [12]. Our study extends the description of the role of NETs in the host response to Gram-negative sepsis caused by *B. pseudomallei* in both clinical melioidosis and a murine model of melioidosis*.* We found that the key NET components, nucleosomes and neutrophil elastase, were both elevated in clinical melioidosis. *In vitro* observations supported the notion that neutrophils are the source of these proteins and isolated human neutrophils infected with *B. pseudomallei* released NET-related markers in a time- and dose-dependent manner. We further showed that NETs killed *B. pseudomallei* in an *in vitro* human neutrophil infection model, although in a murine model of experimental melioidosis, in which DNase was used to disrupt NET formation, no protective role for NETs in the host defense against *B. pseudomallei* infection could be demonstrated.

Our data corroborate a previous report in which it was shown that bactericidal NETs were released from human neutrophils in response to *B. pseudomallei* in an *in vitro* model [46]. In addition, we recently showed that inhibition of endogenous activated protein C, which exerts cytoprotective effects on the endothelium by cleavage of histones [47], leads to excessive nucleosome release in BALF during murine experimental melioidosis [48]. We now demonstrate that there is an abundant release of NET-related markers in plasma from melioidosis patients. *In vitro* experiments demonstrated that NET-related markers are released by neutrophils and exhibit NET-dependent antibacterial activity upon infection with *B. pseudomallei*.

Surprisingly, we found that NETs played no role in bacterial clearance in our experimental mouse model of sepsis. In DNase-treated mice, circulating nucleosomes were decreased but there was no difference in cellular influx nor bacterial counts and cytokine release. This also argues against a role for nucleosomes (which can become harmful to the host when released in excessive amounts [25,26]) as significant endogenous danger signals in melioidosis. This correlates with our clinical finding that the concentration of circulating NET markers in melioidosis patients was not different between survivors and non-survivors on day of admission. We did however find a difference between survivors and non-survivors on day 7, which is in line with our previous finding that circulating nucleosomes correlated with severity of the inflammatory response and outcome in a cohort of children suffering from meningococcal sepsis [49].

ELISAs detecting nucleosomes have proven to be a good indicator of NET release in patients [43] but are not specific for nucleosomes released by neutrophils. We therefore cannot exclude that nucleosomes released into the circulation by other cell types, such as endothelial and parenchymal cells, are detected as well. However, the positive correlation with neutrophil elastase together with the marked up-regulation of genes encoding the central NET-associated proteins does suggest that neutrophils are the main source of the detected nucleosomes. Although *B. pseudomallei* can induce NET release, NETs are predominantly a mechanism for clearing extracellular pathogens, and *B. pseudomallei* may escape NETosis by hiding intracellularly [50,21,51]. Indeed, it was found that both the type 3 secretion system (an important virulence factor of *B. pseudomallei*) and the bacterial capsule may play a role in evading NETs [46]. Furthermore, one could hypothesize that like *S. aureus* [52], *B. pseudomallei* is able to cause leukocyte toxicity by converting NETs into a bioproduct thereby inducing macrophage apoptosis. However, this mechanism would be unfavorable for *B. pseudomallei* as it is already known that macrophage destruction is one of the host immune defense strategies to restrict intracellular growth of *B. pseudomallei* [2]. Another possibility could be that plasma from patients with severe sepsis induces platelet-neutrophil interactions in a TLR4-dependent fashion leading to the production of NETs [53,22], whereas TLR4 does not seem to play an important role in the clearance of *B. pseudomallei* [2,41].

At least a third of all patients with melioidosis have diabetes mellitus as a predisposing factor [3,1,2]. An explanation for this increased susceptibility was sought in the function of neutrophils from diabetic patients, which displayed impaired phagocytosis of *B. pseudomallei*, reduced migration, and the inability to produce an oxidative burst to kill intracellular bacteria [54]. Furthermore, it was shown that isolated neutrophils from patients with diabetes mellitus released less extracellular DNA upon *in vitro* stimulation with *B. pseudomallei*, which was associated with a reduced bacterial killing capacity when compared to neutrophils derived from healthy controls [46]. However, in our study, we did not observe a difference in the release of NET-related markers between diabetic and non-diabetic melioidosis patients. The combination of clinical data and data from our murine model argues against an important role for the NETs in melioidosis. Our data also suggest that impaired NET release does not provide an explanation for the increased susceptibility to *B. pseudomallei* infection in patients with diabetes.

NET formation may also injure the host [21]: our finding that DNase-treated mice have diminished hepatocellular injury after infection is consistent with previous reports in which mice deficient in NET components have decreased hepatocellular inflammation compared to controls in an *E. coli*-induced sepsis model [55,22].

## Conclusion

We have shown that *B. pseudomallei* is a potent inducer of NETosis, as reflected by increased concentrations of NET-related components in patients with melioidosis. Although NETs exhibit antibacterial activity against *B. pseudomallei in vitro*, NET formation does not protect against bacterial dissemination and inflammation in a murine model of *B. pseudomallei* infection. This correlates with our clinical finding that levels of NET components do not correlate with mortality.

## Additional files

Additional file 1: Table S1. Characteristics of study subjects. Additional file 2: Table S2. Proteins that localize to NETs in peripheral leucocytes from patients with melioidosis. Additional file 3: Figure S1.
*B. pseudomallei* is as potent as *S. aureus* in the induction of NETosis. Isolated human neutrophils were stimulated with either *Burkholderia pseudomallei* (Bps) or *Staphylococcus aureus* (SA) for 4 h after which elastase (A) and extracellular DNA (B) release were measured. For both bacterial strains, equal MOIs (10^1^) were used. Medium (HBSS−/−) served as negative control and PMA, a known inducer of NETs, as positive control. Extracellular DNA was measured using a Picogreen dsDNA kit. Mean and SDs are shown. *P* value comparing Bps, SA, and PMA to medium control was determined via unpaired *t* test after log transformation of the data. Blood from three to six different healthy human volunteers were used. *P* values *< 0.05, **< 0.01, ***< 0.001 are shown.

## Abbreviations

ALT: alanine transaminase
AST: aspartate aminotransferase
*B. pseudomallei*: *Burkholderia pseudomallei*
BALF: bronchoalveolar lavage fluid
CXCL1/KC: chemokine (C-X-C motif) ligand 1
DAMPS: danger-associated molecular patterns
IFN-γ: interferon-gamma
IL: interleukin
LDH: lactate dehydrogenase
MCP-1/CCL2: monocyte chemotactic protein-1
MOI: multiplicity of infection
NET: neutrophil extracellular traps
PMA: phorbol 12-myristate 13-acetate
*S. aureus*: *Staphylococcus aureus*
TNF-α: tumor necrosis factor-alpha
