# Supplementary material for: Neutrophil extracellular traps in the host defense against sepsis induced by Burkholderia pseudomallei (melioidosis)
Source: Intensive Care Med Exp. 2014 Sep 3;2:21. doi: 10.1186/s40635-014-0021-2 (PMC4678137; doi:10.1186/s40635-014-0021-2)
Supplement: Additional file 1: Table S1. — Characteristics of study subjects. [file 40635_2014_21_MOESM1_ESM.doc]

Additional file 1: Table S1. Characteristics of study subjects

|  | Controls | | Melioidosis | |
| --- | --- | --- | --- | --- |
|  | Blood donors  n=30 | Diabetes patients n=52 | No diabetes  n=10 | Diabetes  n=34* |
| Mean, age, years | 41.5 (37.5-45.4) | 57.5 (54.1-60.9) | 51.6 (40.9-62.3) | 52.9 (49.8-56.0) |
| Male sex | 80.0% (24 of 30) | 34.6% (18 of 52) | 90.0% (9 of 10) | 61.8% (21 of 34) |
| Glucose, mg L^-1^ | 101 (87-117) | 126 (117-136) | 124 (97-159) | 214 (188-244) |
| HbA_1c_, % | 5.8 (5.4-6.3) | 8.2 (7.8-8.5) | 6.0 (5.5-6.5) | 10.6 (9.6-11.7) |
| Mortality |  |  | 0.0% (0 of 0) | 35.2% (12 of 34) |

Age, glucose and HbA_1c_, are reported as mean (95% confidence interval). *This total includes 10 patients were given a new diagnosis of diabetes by admission HbA_1c_ concentration. Additional Table 1 reproduced with permission from Thrombosis and Hemostasis .
