# Supplementary material for: Neutrophil extracellular traps in the host defense against sepsis induced by Burkholderia pseudomallei (melioidosis)
Source: Intensive Care Med Exp. 2014 Sep 3;2:21. doi: 10.1186/s40635-014-0021-2 (PMC4678137; doi:10.1186/s40635-014-0021-2)
Supplement: Additional file 2: Table S2. — Proteins that localize to NETs in peripheral leucocytes from patients with melioidosis. [file 40635_2014_21_MOESM2_ESM.doc]

**Additional file 2: Table S2. Proteins that localize to NETs in peripheral leucocytes from patients with melioidosis**

| **Cellular localization** | **Protein name** | **Gene name** | **Uniprot** | **Differential**  **regulation** | ***P*** |
| --- | --- | --- | --- | --- | --- |
| Granule | Leukocyte elastase | *ELANE* | P08246 | – |  |
|  | Lactotransferrin | *LTF* | P02788 | – |  |
|  | Azurocidin | *AZU1* | P20160 | – |  |
|  | Cathepsin G | *CTSG* | P08311 | – |  |
|  | Myeloperoxiade | *MPO* | P05164 | – |  |
|  | Leukocyte proteinase 3 | *PRTN3* | P24158 | – |  |
|  | Lysozyme C | *LYZ* | P61626 | – |  |
|  | Neutrophil defensin 1 | *DEFA1* | P59665 | – |  |
|  | Neutrophil defensin 3 | *DEFA3* | P59666 | – |  |
| Nucleus | Histone H2A | *H2AFJ* | Q9NV63 | – |  |
|  | Histone H2B | *HIST2H2BE* | Q16778 | up | 2.41 × 10^–6^ |
|  | Histone H2B-like |  | Q3KP43  Q6GMR5 | – |  |
|  | Histone H3 | *HIST2H3A* | Q71DI3 | – |  |
|  | Histone H4 | *HIST1H4A* | P62805 | up | 6.87 × 10^–8^ |
|  | Myeloid cell nuclear differentiation antigen | *MNDA* | P41218 | – |  |
| Cytoplasm | S100 calcium-binding protein A8 | *S100A8* | P05109 | up | 2.18 × 10^–7^ |
|  | S100 calcium-binding protein A9 | *S100A9* | P06702 | up | 1.7 × 10^–4^ |
|  | S100 calcium-binding protein A12 | *S100A12* | P80511 | up | 3.4 × 10^–9^ |
| Cytoskeleton | Actin beta | *ACTB* | P60709 | – |  |
|  | Actin gamma | *ACTG1* | P63261 | down | 3.07 × 10^–5^ |
|  | Myosin-9 | *MYH9* | P35579 | – |  |
|  | Alpha-actinin-1 | *ACTN1* | P12814 | – |  |
|  | Alpha-actinin-4 | *ACTN4* | O43707 | – |  |
|  | Plastin-2 | *LCP1* | P13796 | up | 2.51 × 10^–5^ |
|  | Cytokeratin | *KRT10* | P13645 | – |  |
| Peroxisomal | Catalase | *CAT* | P04040 | down | 1.51 × 10^–3^ |
| Glycolytic enzymes | Alpha-enolase | *ENO1* | P06733 | – |  |
|  | Transketolase | *TKT* | P29401 | – |  |

Gene expression of proteins that localize to the presence of NET-related markers in patients with culture confirmed and septic melioidosis compared to healthy controls . The gene symbols are assigned by the HUGO gene nomenclature committee.
