# Supplementary figures and images for: Neutrophil extracellular traps in the host defense against sepsis induced by Burkholderia pseudomallei (melioidosis)
Source: Intensive Care Med Exp. 2014 Sep 3;2:21. doi: 10.1186/s40635-014-0021-2 (PMC4678137; doi:10.1186/s40635-014-0021-2)

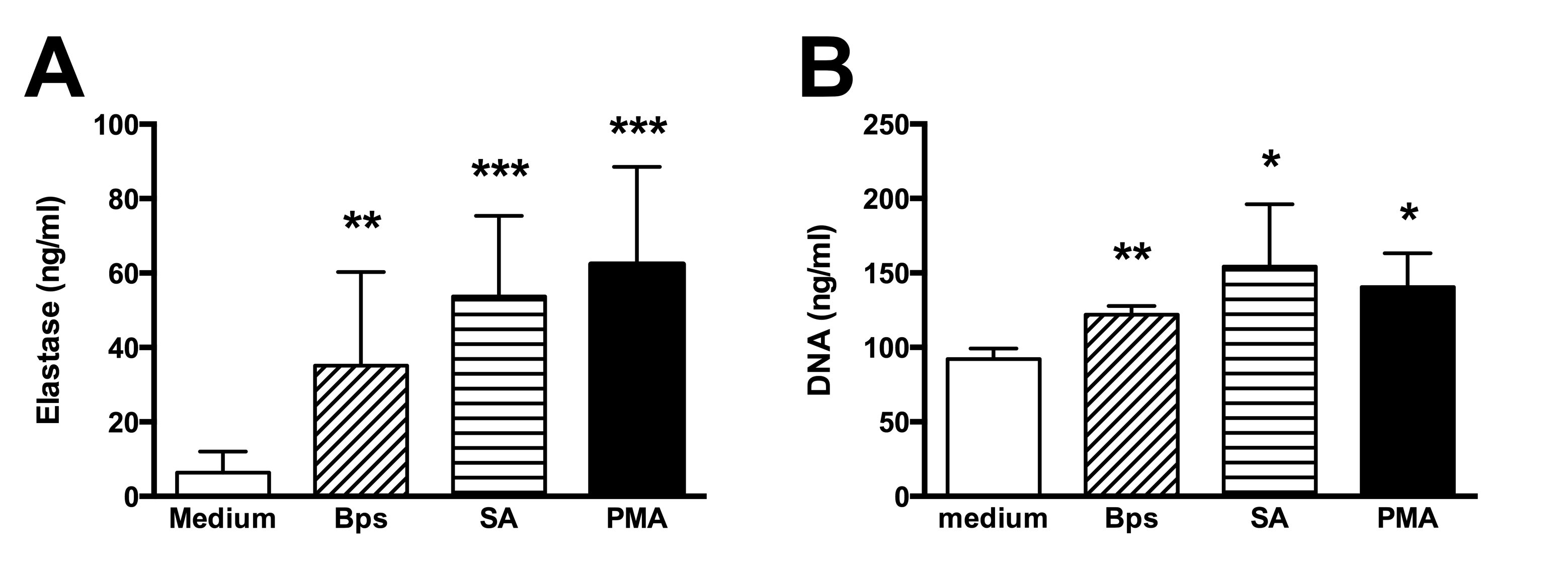

Supplement: Additional file 3: Figure S1. — B. pseudomallei is as potent as S. aureus in the induction of NETosis. Isolated human neutrophils were stimulated with either Burkholderia pseudomallei (Bps) or Staphylococcus aureus (SA) for 4 h after which elastase (A) and extracellular DNA (B) release were measured. For both bacterial strains, equal MOIs (101) were used. Medium (HBSS−/−) served as negative control and PMA, a known inducer of NETs, as positive control. Extracellular DNA was measured using a Picogreen dsDNA kit. Mean and SDs are shown. P value comparing Bps, SA, and PMA to medium control was determined via unpaired t test after log transformation of the data. Blood from three to six different healthy human volunteers were used. P values *< 0.05, **< 0.01, ***< 0.001 are shown. [file 40635_2014_21_MOESM3_ESM.tiff]
